# Comparison of primary total hip replacements performed with a direct anterior approach versus the standard lateral approach: perioperative findings

**DOI:** 10.1007/s10195-011-0144-0

**Published:** 2011-07-12

**Authors:** Vincenzo Alecci, Maurizio Valente, Marina Crucil, Matteo Minerva, Chiara-Martina Pellegrino, Dario Davide Sabbadini

**Affiliations:** 1 Department of Orthopaedics and Traumatology, “San Polo” Hospital, via Galvani, 1, 34074 Monfalcone, GO Italy; 2 Anesthesia and Intensive Care Unit, “San Polo” Hospital, via Galvani, 1, 34074 Monfalcone, GO Italy

**Keywords:** Hip arthroplasty, Surgical technique, Direct anterior approach, Minimally invasive surgery

## Abstract

**Background:**

Given the increasing demand for tissue-sparing surgery, the surgical approach is the subject of lively debate in total hip replacement. The aim of this paper is to compare the efficacy of the minimally invasive direct anterior approach and the standard lateral approach to total hip replacement surgery by observing intra- and perioperative outcomes.

**Materials and methods:**

The authors conducted a retrospective study on a group of 419 consecutive patients undergoing total hip replacement for coxarthrosis. The patients were divided into a first group (A) of 198 patients who had surgery with the standard lateral approach, and a second control group (B) of 221 patients who had the same procedure via the minimally invasive direct anterior approach. Assessment of the two groups considered the following perioperative parameters: length of the surgical procedure, intraoperative complications, intra- and postoperative blood loss, postoperative pain, postoperative nausea and vomiting, length of stay, and type of discharge.

**Results:**

The two groups were homogeneous when compared in relation to mean age, sex and body weight. The minimally invasive direct anterior approach was performed within an acceptable time (89 ± 19 min vs. 81 ± 15 min) and with modest blood loss (3.1 ± 0.9 g/dL vs. 3,5 ± 1 g/dL). Patients experienced less pain (1.4 ± 1.5 NRS score vs. 2.5 ± 2 NRS score), and PONV affected only 5% versus 10% of cases. Times to discharge were shorter (7 ± 2 days vs. 10 ± 3.5 days), and 58.4% versus 11.6% of patients were discharged to home.

**Conclusions:**

In our study, patients treated with a minimally invasive direct anterior approach had a better perioperative outcome than patients treated with the lateral approach. The longer time of surgery for the minimally invasive direct anterior approach may be attributed to the learning curve. Further studies are necessary to investigate the advantages of a minimally invasive direct anterior approach in terms of clinical results in the short and long run.

## Introduction

Hip replacement surgery is considered a reliable and reproducible surgical procedure. It reduces pain and restores the movement of the hip joint, thus improving the quality of life of patients previously impaired by the arthritic process.

Although there is general agreement about the surgical procedure, there is still debate about the approach to be used.

The minimally invasive direct anterior approach restores or improves the patient’s functioning, allowing a return to normal everyday life [1–3]. This surgical approach reduces postoperative pain and length of hospital stay and allows discharge to home [4–6] with a considerable cost reduction [7].

The aim of this paper is to compare the efficacy of the minimally invasive direct anterior approach and the standard lateral approach to total hip replacement surgery by observing intra- and perioperative outcomes in two consecutive cohorts of patients.

## Materials and methods

All the patients gave informed consent prior to being included in the study. As this study was a standard of care, local ethics committee authorization was not required. The study was performed in accordance with the ethical standards of the 1964 Declaration of Helsinki as revised in 2000.

We conducted a retrospective study on two consecutive cohorts of patients undergoing total hip replacement surgery at the Orthopedics and Trauma Unit of Ospedale San Polo in Monfalcone (Gorizia, Italy). Group A patients were treated with Bauer’s standard lateral approach under spinal or general anesthesia; group B was treated with the minimally invasive direct anterior approach under general anesthesia.

All surgical procedures for group A were performed by either of two expert surgeons (VA, MV), whereas all minimally invasive direct anterior approach procedures for group B were performed by one surgeon (VA). VA introduced this approach in our hospital, at the beginning of his learning curve.

The two groups were compared in terms of mean age, sex, body weight and ASA class. Assessment of the two groups considered the following parameters:

1. Length of the surgical procedure
2. Intraoperative complications
3. Intra- and postoperative blood loss
4. Postoperative pain
5. Postoperative nausea and vomiting
6. Length of stay and type of discharge

In the intraoperative phase, we considered the length of the surgical procedure and anesthesia (min), fluids administered (mL of crystalloids, colloids, units of blood and blood products) and incidence of complications (acute bleeding, cardiovascular and respiratory events, late awakening).

In the postoperative period we considered the hemoglobin values on the first and third days compared to preoperative values (mg/dL), length of hospital stay (days), units of packed red blood cells and/or blood products transfused (*n*), and incidence of complications (*n*).

DVT prophylaxis with nadroparin calcium at a standard dose of 3,800 U to be titrated to the patient’s weight was started 12 h before the procedure and continued for 30 days afterwards. Antibiotic prophylaxis was administered using cefazolin 2 g i.v. before the induction of anesthesia and amoxicillin/clavulanic acid 1 g × 3 per day for the following 3 days.

For group A, the surgical technique used was Bauer’s standard lateral approach, whereas the surgical technique used for group B was the minimally invasive direct anterior approach.

**Fig. 1:**
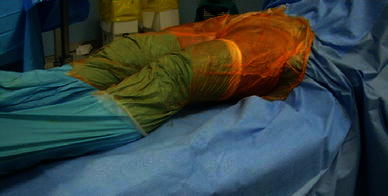
Patient placed in the supine position on a standard orthopedic bed. The sterile field includes both lower limbs

**Fig. 2:**
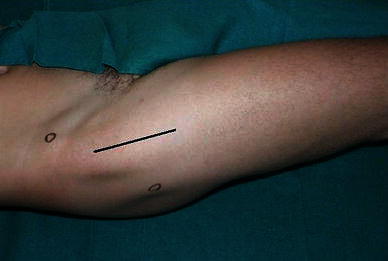
Incision starts 2 cm distal to and 2 cm posterior to the ASIS and continues distally for about 8–10 cm along the straight line joining the lateral edge of the patella

**Fig. 3:**
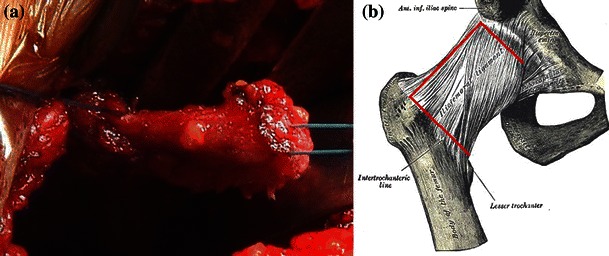
**a**, **b***U*-shaped medially based capsulotomy. The resulting flap is folded over distally to protect the vessels

**Fig. 4:**
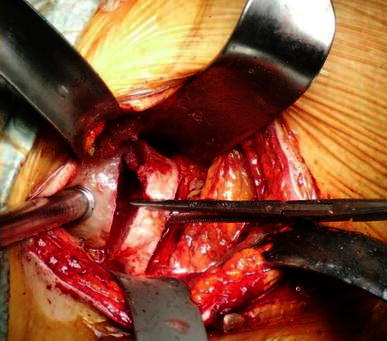
Double osteotomy technique

**Fig. 5:**
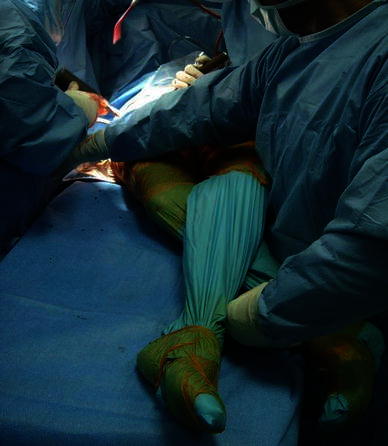
Operated limb adducted below the contralateral limb and rotated outward to allow the proximal metaphysis of the femur to protrude

In the minimally invasive direct anterior approach, the patient was placed in the supine position on a standard orthopedic bed, with the sterile field including both lower limbs (Fig. 1). The landmarks were the anterior superior iliac spine (ASIS) and the lateral edge of the patella. The incision started 2 cm distal to and 2 cm posterior to the ASIS and continued distally for about 8–10 cm along the straight line joining the lateral edge of the patella (Fig. 2). On reaching the fascia, the incision followed the direction of the skin; blunt dissection was then used. A passage was sought between the tensor fasciae latae (lateral) and the sartorius muscles (medial). An incision was made into the perimysium of the rectus femoris muscle, which was medially retracted. Again in a longitudinal direction, an incision was made in the deep fascia and the ascending branches of the lateral circumflex artery were isolated and tied. The capsular plane was prepared by blunt dissection. The iliopsoas muscle was dissected from the capsule and retracted with a dedicated lever. The arthrotomy was performed by making a *U*-shaped medially based capsulotomy, folding the resulting flap distally to protect vessels (Fig. 3a, b). We used a double osteotomy technique, excising a slice of the femur neck (Fig. 4). This technique allowed for a greater mobility of the femoral head and eased extraction. For the preparation of the femoral canal, a critical part of the procedure, the operated limb was adducted below the contralateral one and rotated outward (Fig. 5). This maneuver caused the proximal metaphysis of the femur to protrude, thus allowing the surgeon to work in the canal and apply the stem. At the end of this procedure, a suction drainage was placed and the implant was covered with the capsular flap. Levers were removed and the muscles—m. iliopsoas, m. rectus femoris, m. sartorius, m. tensor fasciae latae—were allowed to return spontaneously to their anatomic positions. The superficial fascia and subcutaneous layer were sutured with absorbable detached sutures (Safil 3-0, Braun Aesculap), whereas metallic sutures were used for the skin.

Active mobilization started on the evening of the procedure. Rehabilitation was started on the first postoperative day, with the aim of allowing patients to walk alone either with or without aids, to walk upstairs and take care of personal hygiene. Patients were instructed to walk without assistive devices when able. The hospital rehabilitation ward and the rehabilitation offered did not change throughout the study. On discharge, patients could choose whether to accomplish their rehabilitation within the hospital, in other dedicated structures, or as outpatients.

The analgesic protocol was based on the administration of opioids, NSAIDs, gastroprotective and antiemetic drugs using an elastomeric pump for 24 h. Pain was measured by the Numeric Rating Scale (NRS) from 1 to 10. This parameter was assessed by the ward nurses twice daily and the anesthesia nurses in charge of the Acute Pain Service twice daily, starting a couple of hours after the end of surgery. Rescue doses of medications were administered accordingly if NRS was >4.

Statistical analysis was done with the aid of the SPSS software package (SPSS, Chicago, IL, USA), applying the independent samples *t*-test for normal variable parameters (age, weight, crystalloids, Hb values, length of stay) and the Mann–Whitney *U* test for non-normally distributed variables (colloids, reinfusion drains, total drain, NRS score). Pearson’s chi-square test was used to compare categorical data (ASA status) and Fisher’s exact test was used to compare dichotomous variables (sex, transfusions, outcome). Where appropriate, ranges and interquartile ranges are indicated. A *P* value of <0.05 was considered to be significant.

## Results

In all, 419 consecutive patients undergoing hip replacement surgery between July 2006 and June 2009 were considered. Group A patients (*n* = 198) were treated with a standard lateral approach and spinal anesthesia (July 2006–December 2007), and were compared to group B patients (*n* = 221) treated with a minimally invasive direct anterior approach and general anesthesia (January 2008–June 2009).

The two groups were similar in mean age, weight and sex. ASA status differed significantly in the two groups (Table 1).

**Table 1:** Population characteristics

	Group A (*n* = 198)		Group B (*n* = 221)		*P*
Age (years ± SD)	70.15 ± 9.6 (42–93)		70.7 ± 8.2 (43–89)		0.52
Weight (kg ± SD)	74.5 ± 13 (46–112)		74.9 ± 13 (42–113)		0.77
Sex (male/female)	75/123		100/121		0.137
ASA status ASA I	23	11.6%	40	18.2%	0.035
ASA II	127	64.1%	146	66%	
ASA III	48	24.3%	35	15.8%	

Statistical analysis: ASA status: Pearson’s chi-square test

Sex: Fisher’s exact test (two-sided)

Age, weight: independent sample *t* test

The mean lengths of surgery for the procedures were statistically significantly different (*P* < 0.05), with the direct anterior procedure lasting an average of 8 min more than the lateral approach (Table 2). We had a longer duration of anesthesia in patients in group B, all of whom were given general anesthesia: this time was considered to stretch from induction to awakening. Spinal anesthesia was given to 77.3% of the patients in group A. Duration of anesthesia was considered to last from the subarachnoid injection of the local anaesthetic to the end of the surgical procedure.

**Table 2:**
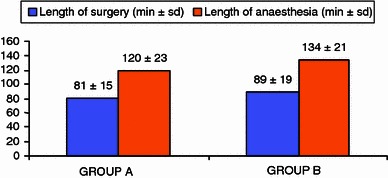
Length of surgery and anaesthesia

Intraoperative complications resulted in admission to the intensive care unit for 4.5% of the patients in group A (*n* = 9) and 7.2% of the cases in group B (*n* = 16). Indications for ICU admission were postoperative monitoring (group A *n* = 2; group B *n* = 7), late awakening (group B *n* = 8), cardiocirculatory complications (group A *n* = 6; group B *n* = 1), and hemorrhage (group A *n* = 1). Transfer to the ward occurred on the same day as the procedure or on the first postoperative day.

We observed the following orthopedic complications in group A: 1 case of nerve injury, 1 case of DVT treated pharmacologically, 3 cases of hematoma treated with incision and drainage. In group B we observed 2 cases of hematoma treated with incision and drainage and 2 fractures of the greater trochanter that did not require changes in the surgical procedure, nor any surgical treatment. There were no cases of dislocation or infection in either group.

The fluid volumes infused during the different procedures were statistically significantly different. Infusions of crystalloids (+490 mL/patient), colloids (+174 mL/patient) and the intraoperative administration of autologous and homologous packed red blood cells were significantly higher in patients treated with the lateral approach (7.5% vs. 1.8%) (Table 3).

**Table 3:** Intraoperative fluid administration

	Group A	Group B	*P*
Crystalloids (mL ± SD)	2,190 ± 700 (2,092–2,288)	1,700 ± 570 (1,625–1,775)	<0.0005
Colloids mL (mean, interquartile range)	301, 500 (0–1,500)	127, 0 (0–1,000)	<0.0005
Homologous blood (no patients)	8	3	
Autologous blood (no patients)	7	1	
Blood transfusions (homologous + autologous)	7.5%	1.8%	0.008

Statistical analysis: crystalloids: independent sample *t* test

Colloids: Mann–Whitney *U* test

Transfusions: Fisher’s exact test (two-sided)

Hemoglobin values were recorded on the first and third day after the procedure and compared with preoperative values. Hb values were significantly higher in patients treated with the direct anterior approach (10.6 g/dL vs. 9.7 g/dL), with a statistically significant difference of 3.5 g/dL versus 3.1 g/dL in favor of group B (Table 4).

**Table 4:** Hb values: preoperative, first and third postoperative days (Hb g/dL ± SD)

	Group A	Group B	*P*
Preoperative	13.3 ± 1.3 (13.1–13.5)	13.7 ± 1.4 (13.5–13.9)	0.001
Day 1	9.7 ± 1.3 (9.5–9.9)	10.6 ± 1.3 (10.4–10.8)	<0.0005
Day3	9.7 ± 1.0 (9.6–9.8)	10.1 ± 1.3 (9.9–10.3)	0.001
Δ (Hb PRE − Hb1)	3.5 ± 1.0 (3.4–3.6)	3.1 ± 0.9 (3.0–3.2)	<0.0005

Statistical analysis: independent samples *t* test

Blood transfusions in the postoperative period were significantly more frequent in group A than in group B (40% vs 19.5% of patients) (Table 5). The blood volume collected from drains and reinfused was greater in group A (275 mL vs. 271 mL), as was the total volume of blood from drains (380 mL in group A; 189 mL in group B (Table 5).

**Table 5:** Postoperative transfusions and reinfusions, surgical drains

	Group A	Group B	*P*
Homologous blood (no patients)	55	28	
Autologous blood (no patients)	30	16	
Blood transfusions (homologous + autologous)	40%	19.5%	<0.0005
Reinfusion drain (median, interquartile range in mL)	275, 250 (0–1,150)	271, 250 (0–1,000)	0.014
Total drain (median, interquartile range in mL)	380, 385 (0–1,400)	189, 300 (0–1,250)	<0.0005

Statistical analysis: transfusions: Fisher’s exact test (two-sided)

Reinfusion drain, total drain: Mann–Whitney *U* test

The mean NRS score on the first postoperative day was 2.5 for group A and 1.4 in group B. PONV affected 10% of group A and only 5% of cases in group B.

Length of hospital stay differed significantly (10 days for group A; 7 days for group B). Table 6 shows the results according to time and outcome of discharge. By postoperative day 4, 22.6% of the patients treated with the anterior approach were discharged, and about 87% were discharged by day 7. Only 1% of the patients treated via the lateral approach were discharged by day 4 and 10% by day 7. Observing the outcome of discharge, 88.4% of group A patients were discharged to a dedicated postoperative rehabilitation center and 11.6% were discharged home. In group B, 58.4% of patients were discharged to a dedicated postoperative rehabilitation center and 41.6% were discharged home.

**Table 6:** Discharge—timing and outcome

	Group A	Group B	*P*
Length of stay (days ± SD)	10 ± 3.5 (9.5–10.5)	7 ± 2 (6.7–7.3)	<0.0005
Outcome on discharge (nursing home/patient’s home)	175/23	129/92	<0.0005

Statistical analysis: length of stay: independent samples *t* test

Outcome: Fisher’s exact test

## Discussion

In recent years, minimally invasive hip replacement surgery has become increasingly popular [8]. However, certain minimally invasive surgical procedures have been characterized by a higher complication rate than conventional techniques [9–11].

The direct anterior approach is a modification of the Smith–Petersen approach, as only the distal part of the anterior superior iliac spine is used. This technique became routine practice for Judet and Judet in 1947 [12, 13] with the introduction of a dedicated trauma table. More recently, Matta transformed the surgical procedure into a minimally invasive procedure in which the patient is also positioned on a dedicated trauma table [4, 14]. Currently, more and more surgeons perform the minimally invasive direct anterior approach without a dedicated trauma table [1, 5, 15]. This variant offers a number of advantages: it does not require the presence of personnel trained to perform trauma table maneuvers; furthermore limb length, implant stability, and movement of the operated hip can be checked more readily, as the lower limbs are free.

In our experience, the objectives set for the minimally invasive anterior approach were achieved in the perioperative period compared to the control group treated with lateral surgical access. The two cohorts treated with different surgical and anesthesiology protocols obtained significantly different results for the parameters considered as measures of outcome.

The two groups were similar in terms of sex, age and weight. The difference in ASA status distribution (group A included a greater number of ASA III patients, whereas group B had a greater incidence of ASA I patients) can be attributed to a technique selection bias due to the combination of anterior approach–general anesthesia. A higher ASA score is associated with higher perioperative risks, supporting the choice of a locoregional technique (and lateral approach), even after the introduction of the anterior direct approach.

The mean procedure time from incision to suture was on average longer with the anterior approach. This could be consistent with our learning curve; however, when the data were considered separately for each year, the mean remained higher in the second year. This may be related to the complexity of the minimally invasive anterior approach, which requires greater skill in the various steps.

All group B patients were given general anesthesia, whereas over 75% of group A patients received locoregional (subarachnoid and epidural) anesthesia. We chose general anesthesia to accommodate muscle relaxation requirements, to avoid minimizing the surgical field and causing potential damage to the soft tissues under traction [16]. In our experience, an optimal level of relaxation cannot be obtained using locoregional techniques. Muscle relaxation is essential during the femoral stump preparation stages. Although we recognize the theoretical adequacy of locoregional anesthesia, we prefer general anesthesia with a deep muscular relaxation achieved using high-dose neuromuscular blockers. The comparison between locoregional and general anesthesia must consider the progress made in the field of thromboprophylaxis and intraoperative monitoring of the new rapid-clearance or short half-life drugs introduced in recent years [17].

In the intraoperative period, the incidence of complications requiring admission to the intensive care unit (ICU) was low in both cases, although higher in cases treated via the anterior approach. In 50% of all cases (i.e. 8), ICU admission was necessary for delayed awakening (difficulties resuming spontaneous respiration/respiratory insufficiency/reduced muscle tone) related to the administration of general anesthesia with muscle relaxation up to the end of the procedure. The incidence of major orthopedic complications was low in both groups (A 5/198; B 4/221). Greater trochanter fracture occurred in two patients in group B. This is a typical complication occurring in the minimally invasive direct anterior approach, related to an insufficient release of the capsule [5]. In our study, this complication occurred in only two cases among the first 50 patients treated with this approach. We considered 50 patients treated to be a reasonable number to accomplish our learning curve.

Lower volumes of fluids were infused during the procedure in patients treated in a minimally invasive approach (crystalloids −22.4% per patient; colloids −57.8% per patient) compared to group A, despite the longer anesthesia and procedure times. This can be attributed to the lower intraoperative blood loss associated with the minimally invasive technique and the use of general anesthesia, which may have resulted in greater intraoperative hemodynamic stability. Macfarlane’s review considers that blood losses are lower using locoregional techniques for anesthesia [18]. The lower losses we observed in group B, despite general anesthesia, support the hypothesis that surgery is mainly responsible for our reduced losses.

Significantly lower blood losses are reflected in smaller changes in pre- and postoperative hemoglobin values, less blood drained, and lower volumes of blood transfusions required. Hemoglobin values, which were slightly higher at baseline in anterior approach patients, dropped to a lesser extent in the postoperative period, despite remaining constant between the first and third days in both cohorts. The anterior approach has therefore allowed us to reduce postoperative homologous and autologous blood transfusions by half. The lower blood loss was also accounted for by the lower volume of blood drained (reinfusion −16.9%; total volume from drain −52%).

Regardless of the analgesic protocol used by the anesthesiologists, postoperative pain was well controlled, with a further reduction in the NRS score of patients undergoing the minimally invasive procedure. The differences in the various analgesic protocols used, often dictated by patient characteristics or anesthesiologist preference, do not allow for reliable assessment of the efficacy of each one in relation to the NRS score.

The length of hospital stay was on average reduced from 10 to 7 days. This is significant in relation to both the minimally invasive technique, which was effective at reducing pain and complications that could require prolonged hospital stays, and the anesthetic technique used. In fact, despite the use of general anesthesia, the use of short half-life drugs does not delay recovery and the start of rehabilitation. Faster recovery allowed for the rapid achievement of rehabilitation goals, reducing the need for a longer hospital stay. Considering the level of autonomy attained by patients in a few days, discharge home was safely feasible.

In conclusion, given greater awareness of the advantages offered by the minimally invasive direct anterior approach, we have tried to validate a minimally invasive surgical technique as a safe and efficacious means of reducing morbidity and accelerating functional recovery. Improvements in the surgical technique and in perioperative anesthesia and analgesia protocols are of fundamental importance for the therapeutic success of the total hip replacement procedure. Further studies are needed to evaluate advantages of the minimally invasive direct anterior approach through analysis of clinical results in the short and long term.
